# Inducible and tunable gene expression systems for *Pseudomonas putida* KT2440

**DOI:** 10.1038/s41598-021-97550-7

**Published:** 2021-09-10

**Authors:** Chandran Sathesh-Prabu, Rameshwar Tiwari, Doyun Kim, Sung Kuk Lee

**Affiliations:** 1 School of Energy and Chemical Engineering, Ulsan National Institute of Science and Technology (UNIST), Ulsan, 44919 Republic of Korea; 2 Department of Biomedical Engineering, Ulsan National Institute of Science and Technology (UNIST), Ulsan, 44919 Republic of Korea; 3 Department of Energy Engineering, Ulsan National Institute of Science and Technology (UNIST), Ulsan, 44919 Republic of Korea

**Keywords:** Expression systems, Metabolic engineering

## Abstract

Inducible and tunable expression systems are essential for the microbial production of biochemicals. Five different carbon source- and substrate-inducible promoter systems were developed and further evaluated in *Pseudomonas putida* KT2440 by analyzing the expression of green fluorescent protein (GFP) as a reporter protein. These systems can be induced by low-cost compounds such as glucose, 3-hydroxypropionic acid (3HP), levulinic acid (LA), and xylose. 3HP-inducible HpdR/P_*hpdH*_ was also efficiently induced by LA. LvaR/P_*lvaA*_ and XutR/P_*xutA*_ systems were induced even at low concentrations of LA (0.1 mM) and xylose (0.5 mM), respectively. Glucose-inducible HexR/P_*zwf1*_ showed weak GFP expression. These inducer agents can be used as potent starting materials for both cell growth and the production of a wide range of biochemicals. The efficiency of the reported systems was comparable to that of conventional chemical-inducible systems. Hence, the newly investigated promoter systems are highly useful for the expression of target genes in the widely used synthetic biology chassis *P. putida* KT2440 for industrial and medical applications.

## Introduction

The production of biobased bulk chemicals from renewable bioresources could minimize the negative impacts of conventional chemical-based productions on the environment and the challenges posed by depleting resources of natural petrochemicals^1^. *Pseudomonas putida* strain KT2440 is a prominent metabolic engineering and synthetic biology chassis for industrial and medical applications because of its robustness and metabolic versatility^2–5^. Recently, a library of synthetic promoters by modifying − 35, − 10, and UP-elements (upstream sequences to − 35) as well as different ribosomal binding sites have been developed for *P. putida* strain KT2440^6^. These promoters constitutively induced the expression of a fluorescent reporter gene with a different strength of protein expression level. In general, constitutive expression systems are detrimental to cells due to uncontrolled production of target protein(s) and structural instability^7^. Nevertheless, tuning of gene expression is essential to optimize the metabolic pathways for achieving high product titers, yields, and productivity^8^.Calero and coworkers characterized different inducible promoters using a set of broad-host-expression vectors in *P. putida* KT2440^9^.

Moreover, a variety of native (P_*m*_, P_*sal*_, P_*alkB*_, P_*u*_, and P_*xylA*_) and heterologous (P_*araB*_, P_*rhaB*_, P_*trc*_, P_*lac*_, P_*tac*_, P_*tet*_, P_*T7-lac*_ and P_*lacUV5*_, P_*mekA*_, P_*mtlE*_, P_*chnB*_, and *P*_*DB3*_) inducible expression systems have been demonstrated in *P. putida* strains^9–11^. These induction systems use a wide range of inducing chemicals such as 3-methylbenzoate, 3-methylbenzyl alcohol, dicyclopropylketone, methyl ethyl ketone, *m*-toluate, salicylate, *n*-octane, 3-chloro-4-hydroxyphenylacetic acid, cyclohexanone, rhamnose, arabinose, xylose, mannitol, *p*-cumate, anhydrotetracycline, and isopropyl-β-D-1-thiogalactopyranoside (IPTG) with varying degrees of success^9–13^. In addition, temperature inducible^14^ and quorum sensing promoter systems^15,16^ were employed in *P. putida* strains. Induction of gene expression by the addition of chemical inducers, such as IPTG, is considered the most efficient method^17^. However, several types of chemical inducers are not amenable to industrial scale-up because of their toxicity and cost^17^. Thus, the use of inexpensive carbon sources or substrates as inducers could be considered as a feasible strategy for the large-scale production of biochemicals.

To increase the strain applicability, we aimed to develop a set of promoters that could be regulated either by a carbon source or substrate in *P. putida* KT2440. The systems constructed here could solve the limitations encountered with other inducible or constitutive expression systems^18,19^. In this study, we focused on evaluating the efficiency of commonly used and naturally abundant carbon sources, including glucose (Glu), levulinic acid (LA), and xylose (Xyl), on the induction of the expression of a green fluorescent protein (GFP) as a quantitative reporter of relative promoter activity. Furthermore, the cross-reactivity of different inducers with promoter systems and the intactness of the promoter systems were analyzed. These low-cost substrates are already used as potent starting materials for both cell growth and the production of a wide range of biochemicals. Although the microbial production of industrially relevant biochemicals using Glu- and LA-inducible expression systems has been previously reported^20,21^, the promoters have not been characterized in detail.

To our knowledge, this is the first report describing the characterization of substrate-inducible and tunable promoter systems for *P. putida* KT2440 at the single-cell level to broaden their applicability.

## Results and discussion

**Figure 1:**
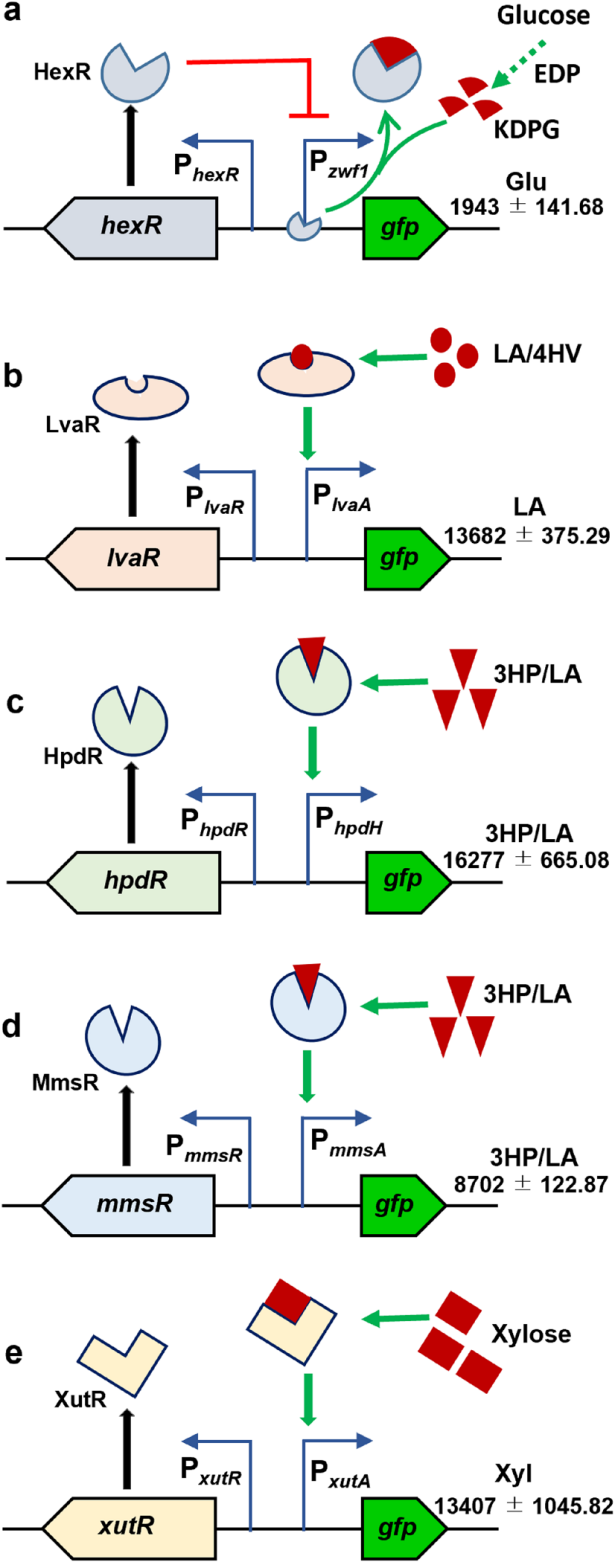
Schematic representations of the activation of the substrate-inducible promoter systems. (**a**) activation of the HexR/P_*zwf1*_ system by glucose through the EDP effector molecule KDPG; (**b**) activation of the LvaR/P_*lvaA*_ system by LA or 4HV; (**c**) activation of the HpdR/P_*hpdH*_ system by 3HP or LA; (**d**) activation of the MmsR/P_*mmsA*_ system by 3HP or LA; (**e**) activation of the XutR/P_*xutA*_ system by xylose. The numerical values (mean ± standard deviation) and the inducer(s) represent the maximum normalized fluorescence intensity of the corresponding system with the respective inducer(s). Both 3HP and LA (3HP/LA) showed the maximum level of expression (*P* > 0.05) in (**c**) and (**d**). EDP, Entner–Doudoroff pathway; KDPG, 2-keto-3-deoxy-6-phosphogluconate; *gfp*, green fluorescent protein; Glu, glucose; LA, levulinic acid; 4HV, 4-hydroxyvalerate; 3HP, 3-hydroxypropionic acid; Xyl, xylose.

In the present study, the strength of each promoter was evaluated using a GFP-based reporter assay. The level of GFP fluorescence intensity was linearly correlated with the concentration of the respective inducers added. Therefore, the useful range of inducer concentration and homogeneity (cell populations with similar expression levels) of all expression systems were analyzed using flow cytometry. Furthermore, amicability between promoters/inducers is necessary when different expression systems are used to employ complex metabolic pathways containing multiple genes. Thus, the cross-talk among promoters/inducers was characterized. Here, five different carbon source- and substrate-inducible promoter systems, HexR/P_*zwf1*_ induced by glucose, LvaR/P_*lvaA*_ by LA or 4HV, HpdR/P_*hpdH*_ by 3HP or LA, MmsR/P_*mmsA*_ by 3HP or LA, and XutR/P_*xutA*_ by xylose, were developed (Fig. 1).

### Glu-inducible HexR/P_*zwf1*_ system

**Figure 2:**
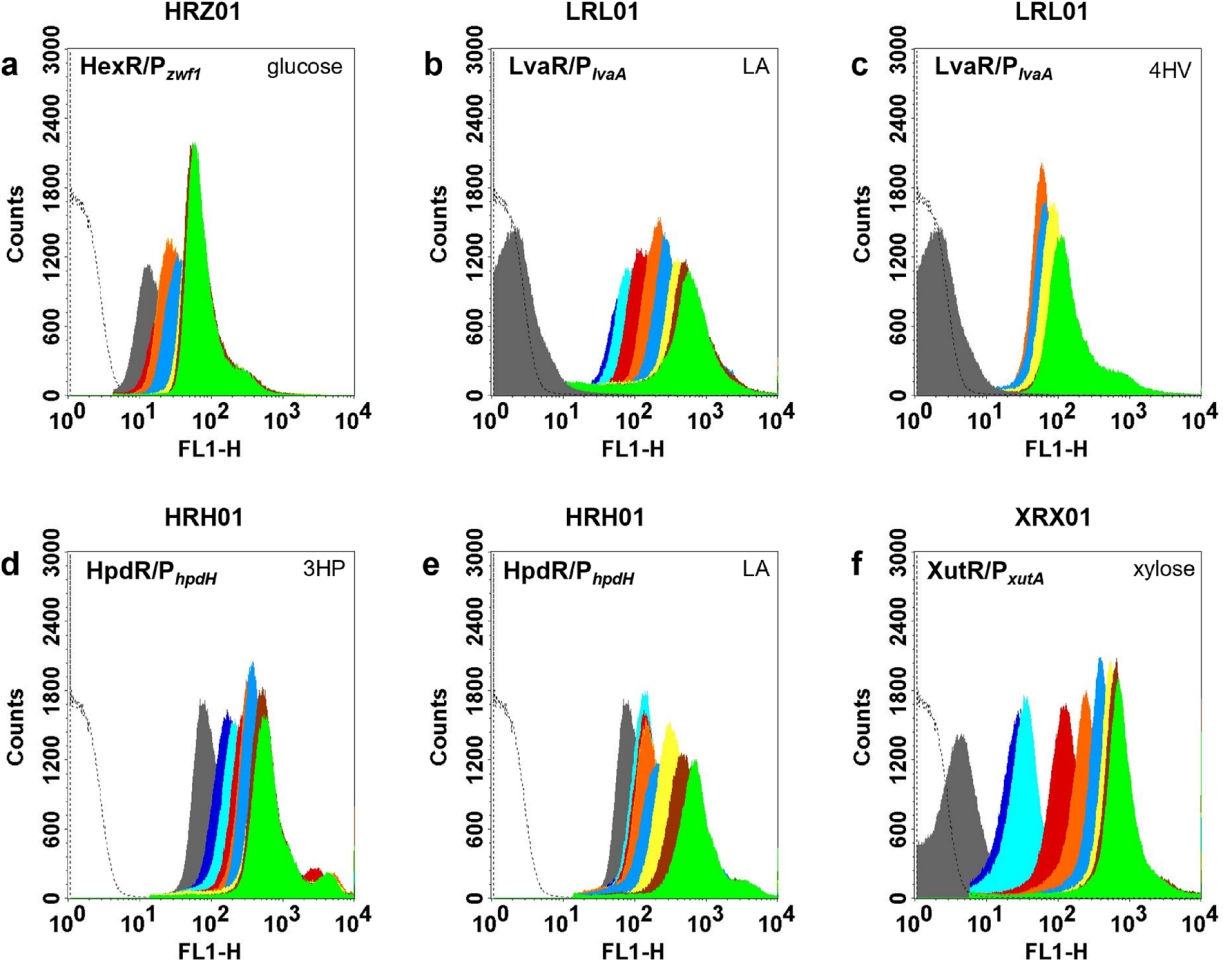
Flow cytometry analysis of substrate-inducible promoter systems. The cultures were incubated with different concentrations of the corresponding inducers. After 8 h of induction, the samples were analyzed for GFP fluorescence using a flow cytometer. HexR/P_*zwf1*_ system induced with glucose (**a**); LvaR/P_*lvaA*_ system induced with LA (**b**) or 4HV (**c**); HpdR/P_*hpdH*_ system induced with 3HP (**d**) or LA (**e**); and XutR/P_*xutA*_ system induced with xylose (**f**).  0 mM;  0.1 mM;  0.2 mM;  0.5 mM;  1 mM;  2 mM;  5 mM;  10 mM;  20 mM;—empty plasmid.

The GFP expression of the strain HRZ01 harboring pHRZ-eGFP^+^ was evaluated in M9Y medium supplemented with different concentrations of Glu (0.5 to 20 mM). As expected, the addition of Glu resulted in a higher fluorescence intensity compared to that in the control condition without Glu (Fig. 2a). The fluorescence intensity increased with increasing concentrations of Glu in the medium. However, no difference was observed when the concentration of Glu exceeded 5 mM. The system was not induced by the addition of Xyl, LA, 4-hydroxyvalerate (4HV), or 3-hydroxypropionic acid (3HP). Furthermore, the addition of either Xyl, LA, 4HV, or 3HP in combination with Glu (10 mM each) did not affect the induction of the HexR/P_*zwf1*_ system (Supplementary Figure S1).

An intermediate of the Entner–Doudoroff pathway (EDP) involves in the activation of the HexR/P_*zwf1*_ system. *P. putida* KT2440 almost exclusively uses the EDP for glucose metabolism, rather than the Embden–Meyerhof–Parnas pathway (EMPP)^5^. The transcriptional repressor HexR in *P. putida* KT2440 negatively regulates the expression of the glucose metabolism genes, such as *gapA* gene and *zwf-pgl-eda* and *edd-glk-gltR2* operons, by binding to consensus sequences found in upstream of target genes^22,23^. This HexR-mediated repression is blocked by the binding of 2-keto-3-deoxy-6-phosphogluconate (KDPG), an exclusive metabolic intermediate of the EDP, to HexR via its sugar isomerase domain, thus inducing the expression of target genes after releasing the repressor (HexR) from the operator sites^22–24^. Therefore, the added Glu was efficiently catabolized via the EDP, thus producing KDPG to bind the HexR protein, resulting in the expression of GFP (Supplementary Fig. 1). A previous study showed that an EDP-activated *Escherichia coli* strain harboring this system efficiently produced 2,3-butanediol (BDO) from Glu with a 71% increased titer compared to the control strain^20^. Moreover, and that the system was comparable to the chemical (anhydrotetracycline) inducible expression system (P_*LtetO-1*_). No considerable difference was observed on the efficiency of both the systems to produce BDO (13.95 g/L vs 14.17 g/L)^20^. Thus, Glu, which is a low-cost carbon source for growth and a starting material in the production of diverse chemicals, could be used as an efficient inducer for the production of target chemicals.

### LA-inducible LvaR/P_*lvaA*_ system

**Figure 3:**
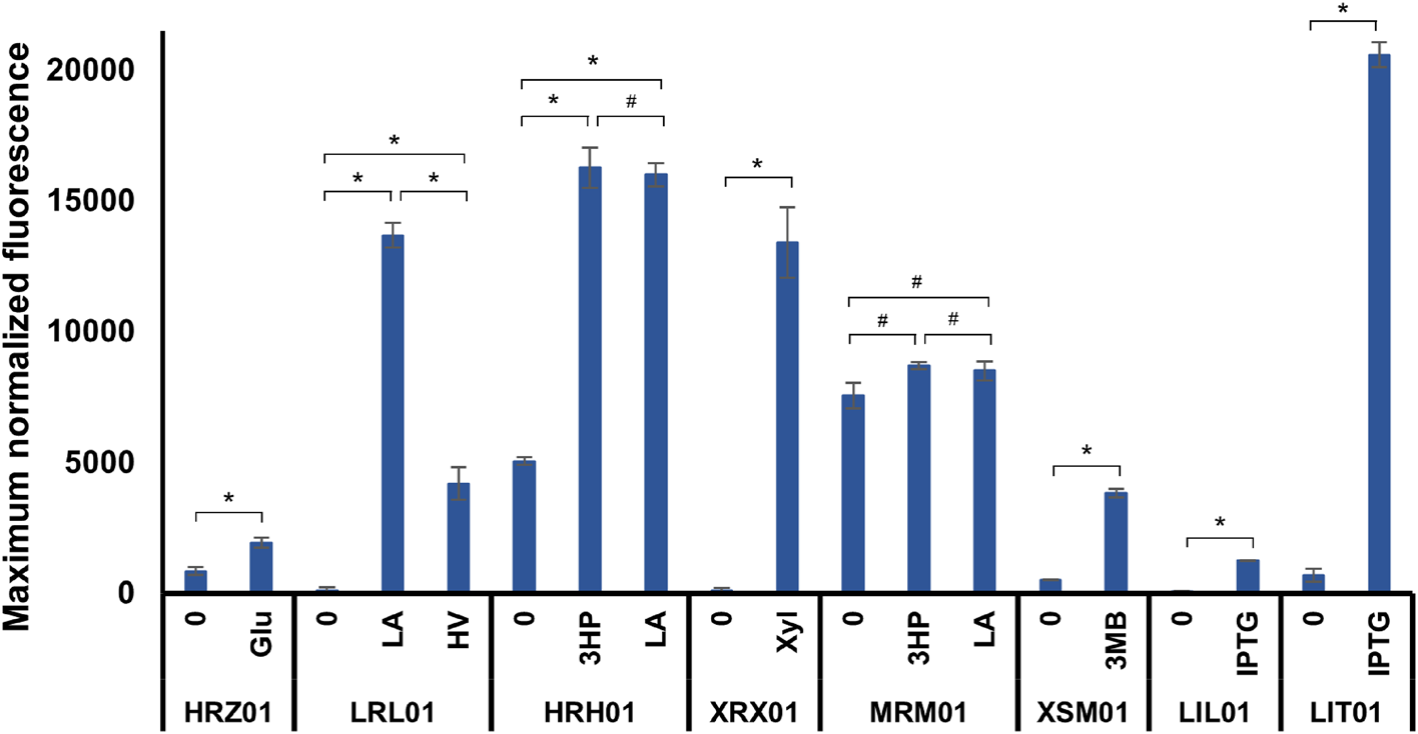
Efficiency of different promoter systems and comparison with chemical inducible systems. Maximum normalized fluorescence was calculated with the saturating concentration of the respective inducers and compared with the control condition (without inducer) and against IPTG (P_*LlacO1*_ and P_*tac*_)- and 3 MB (P_*m*_)-inducible systems. IPTG and 3 MB were used at 0.5 mM. 0, no inducer; Glu, glucose; LA, levulinic acid; 4HV, 4-hydroxyvalerate; 3HP, 3-hydroxypropionic acid; Xyl, xylose; 3 MB, 3-methylbenzoate; IPTG, isopropyl-β-D-thiogalactopyranoside. *P < 0.05; ^#^*P* > 0.05.

The induction of the LvaR/P_*lvaA*_ system was analyzed by estimating the fluorescence intensity of the strain LRL01 harboring pLRL-eGFP^+^ with different concentrations of LA (0.1 to 20 mM) or 4HV (1–20 mM). The addition of LA significantly increased (*P* < 0.05) the fluorescence intensity compared to that in the control (Fig. 2b). The system was inducible even at low concentrations of LA (0.1 mM). Notably, without the inducer, the system was tightly controlled (no leaky expression). The fluorescence intensity increased with increasing concentrations of LA up to 10 mM. Furthermore, the system was also induced by the addition of 4HV, an intermediate of LA catabolism^25^. However, in contrast to LA, 4HV showed non-dose-dependent (all-or-none) expression of the reporter protein (Fig. 2c). In comparison, LA induced a stronger expression than 4HV (Supplementary Figure S2). The LvaR/P_*lvaA*_ system was not induced by Glu, Xyl, or 3HP. However, the expression was slightly affected when the system was induced with LA and Glu (Supplementary Figure S2). This might be due to glucose-mediated carbon catabolite repression^25^. Interestingly, the expression was higher when the system was induced with a mixture of LA and 4HV, compared to that observed with either LA or 4HV alone (Supplementary Fig. 2). When compared with the chemical inducible system (P_*m*_ by 3 MB; strain XSM01), the LvaR/P_*lvaA*_ system (LRL01) showed at least 3.6-fold higher expression levels (Fig. 3). In contrast, the system showed 1.5-fold lower expression than the IPTG-inducible P_*tac*_ system (LIT01). However, the basal expression (without inducer) was sixfold higher in LIT01 than in LRL01.

LA, a five-carbon versatile platform chemical, is identified as one of the “Top-12’ of the most valuable sugar-based building blocks by the United States Department of Energy^26^. It can be obtained through acid-catalyzed dehydration and the hydrolysis of renewable cellulosic biomass especially from cellulose and hemicellulose^27^. LA catabolism, mediated by a seven-gene *lva* operon encoded by *lvaABCDEFG*, was previously elucidated in *P. putida* KT2440^25^. The *lva* operon is regulated by LvaR (transcriptional activator). LvaR activates the expression of downstream genes cloned under the control of P_*lvaA*_ in the presence of LA and/or 4HV. The LvaR/P_*lvaA*_ system exemplifies an interesting phenomenon in which the system can be induced by both the substrate and product. Because it is inexpensive, LA can be used as a starting material to produce a wide range of chemicals, including polymers, plasticizers, fuels, resins, pharmaceuticals, anti-freeze agents, and solvents^27–30^. The *lvaAB-*deleted *P. putida* KT2440 strain produced a maximum of 50 g/L of 4HV with 97% conversion from LA using the LA-inducible LvaR/P_*lvaA*_ system without the addition of any chemical inducers^21^. LA can be used as a carbon source for strain KT2440^21,25^. As the *lva* operon-disturbed strain did not grow on LA, the added LA could not be intrinsically catabolized by the other endogenous system^21,25^. It is noteworthy that in strain KT2440, LA is catabolized to the central metabolites, acetyl-CoA and propionyl-CoA. Thus, the LA-inducible LvaR/P_*lvaA*_ system could be useful for producing a wide range of chemicals from LA.

### LA-inducible HpdR/P_*hpdH*_ system

The fluorescence intensity of strain HRH01 harboring pHRH-eGFP^+^ increased with increasing concentrations of 3HP (Fig. 2d). The addition of Glu or Xyl along with 3HP did not strongly affect induction (*P* > 0.05; Supplementary Figure S3). In addition, the system was not induced by the addition of either Glu or 4HV. However, this system was induced by the addition of LA (Supplementary Figure S3). Therefore, the efficiency of the system was further evaluated using LA as an inducer (Fig. 2e). Compared with the HpdR/3HP system (HRH01 induced by 3HP), the HpdR/LA system (HRH01 induced by LA) was not very sensitive to the lowest concentration of LA (< 1 mM). However, the fluorescence increased with increasing concentrations of LA in a dose-dependent manner (Supplementary Fig. 4). It may be advantageous to have an HpdR/P_*hpdH*_ system that can be induced with a low-cost substrate, such as LA. To our knowledge, this is the first description of the induction of an HpdR/P_*hpdH*_ system by LA.

3HP is a C3 biotechnologically important platform chemical. It is used as a potent building block for deriving both commodity and specialty chemicals^26^. It can be used as a carbon and energy source by *Pseudomonas* species, possessing 3HP catabolic pathways^31,32^. Zhou and coworkers reported that the 3HP-catabolism in *P. denitrificans* is activated by LysR-type transcriptional regulators in the presence of 3HP^32^. In *P. putida*, the 3HP catabolic genes and their respective transcriptional regulators (HpdR or MmsR) are arranged in two operons: (i) *hpdH* and its regulator *hpdR* in one operon, and (ii) *mmsA* and *mmsB*, and their regulator *mmsR* in another^31^. Upon binding of a 3HP molecule to HpdR, the resulting conformational changes in DNA enhance RNA polymerase binding in the promoter sequence of the downstream genes, thus allowing the transcription of the genes. The orthogonality of the P_*hpdH*_ and P_*mmsA*_ of *P. putida* KT2440 was demonstrated in *E. coli* and *Cupriavidus necator*^31^. Moreover, the study of cross-reactivity of some chosen compounds that are structurally similar to 3HP with the P_*hpdH*_ system showed at least 5 to 30% relative induction level^31^. In the present study, LA (10 mM) exhibited around 82% relative induction level of the natural inducer 3HP (10 mM) with the HpdR/P_*hpdH*_ system (Supplementary Figure S4). The relative induction level reached 99.8% with 20 mM of LA. This is advantageous because the system can be activated by two different potential substrates, C3 (3HP) and C5 (LA), which can be used as the starting materials to produce a wide range of chemicals. Furthermore, as both substrates can be completely catabolized by *P. putida* KT2440, a complete deletion or disruption of the catabolic pathways for LA and/or 3HP could be considered to achieve higher performance of the synthetic pathways.

Moreover, the induction of the MmsR/P_*mmsA*_ closely related promoter system was also analyzed by estimating the fluorescence intensity of the constructed strain MRM01 with different concentrations of LA or 3HP (1–20 mM). Strain MRM01 did not show any considerable increase in fluorescence intensity when induced with LA or 3HP compared to the control. The system was not tightly controlled, and a highly leaky regulation was observed (Supplementary Figure S5).

### Xyl-inducible XutR/P_*xutA*_system

The induction of P_*xutA*_ with XutR was analyzed by estimating the fluorescence intensity of the strain XRX01 cultivated in M9Y medium supplemented with different concentrations of Xyl (0.1–20 mM). XutR/P_*xutA*_ was strongly induced by the addition of Xyl (Fig. 2f); even at concentrations as low as 0.5 mM, a fluorescence intensity four-fold stronger than that in the control was observed (Supplementary Figure S6). The system was tightly controlled (no leaky expression, *P* < 0.01). Moreover, a dose-dependent fluorescence intensity was observed for up to 10 mM Xyl. Interestingly, the system was not induced by other C5 (arabinose) or C6 (Glu and mannose) sugars or other commonly tested inducers (LA, 4HV, and 3HP) (Supplementary Fig. 6). This is advantageous when a synthetic pathway necessitates the use of multiple genes under the control of different inducers to avoid cross-reactivity of the system, thus achieving smooth regulation of the metabolic pathways. In the present study, XutR/P_*xutA*_ showed a slower induction compared to that of the other systems studied here (Supplementary Fig. 6). However, the system showed the least basal expression (not leaky) and strong expression on induction, suggesting the non-specific transport of Xyl into the cell. The slower induction might be due to the absence of specific Xyl transporters, such as XylE found in *E. coli*, in *P. putida* KT2440^33^. A similar shortcoming was observed for other sugars, such as arabinose and rhamnose, for P_*araE*_ or P_*rhaB*_ in the strain KT2440^9^.

As *P. putida* KT2440 is not capable of catabolizing xylose, three different xylose utilization pathways, such as xylose isomerase, Dahms, and Weimberg pathways were implemented in KT2440^34,35^. These pathways catabolize xylose into various intermediates and introduce them into the pentose phosphate pathway, glycolysis, or tricarboxylic acid (TCA) cycle, respectively^33–35^. In *P. fluorescens* SBW25, the Xyl catabolic gene *xutA* (encoding xylose isomerase) is regulated by the xylose utilization regulator XutR^36^. XutR functions as a dimer. XutR/PA-II binding affinity is enhanced in the presence of Xyl and activates the promoter in a Xyl dose-dependent manner^36^. Thus, by engineering the strain for the catabolism of Xyl, a wide range of chemicals including amino acids, rhamnolipids, and xylitol could be produced^35^. After Glu, Xyl is the second most abundant sugar in nature and is a potential feedstock for the microbial production of biochemicals. Cellulosic hydrolysate can be efficiently used as it generally contains glucose and xylose in the major portion^37^. Therefore, the Xyl-inducible system could serve as an effective inducible expression system to produce various chemicals from this low-cost carbon source/substrate.

### Intercomparison of different substrate-inducible expression systems

Intercomparison results indicated that the maximum levels of expression were achieved by HpdR/P_*hpdH*_ with the addition of 3HP and LA, XutR/P_*xutA*_ with the addition of Xyl, and by LvaR/P_*lvaA*_ with the addition of LA (Fig. 3). The saturating concentration of inducers for the highest expression level varied among the promoter systems: 5 mM Glu for HexR/P_*zwf1*_, 10 mM LA for LvaR/P_*lvaA*_, 10 mM Xyl for XutR/P_*xutA*_, and 10 mM 3HP or 20 mM LA for HpdR/P_*hpdH*_. The saturating concentration of inducers were found to be within the normal range of widely used concentrations as reported in a comprehensive study on the characterization of inducible promoters using 3 MB (P_*m*_), rhamnose (P_*rhaB*_), arabinose (P_*araB*_), salicylate (P_*sal*_), and IPTG (P_*T7-lac*_ and P_*lacUV5*_) in *P. putida* KT2440^9^. The LvaR/P_*lvaA*_ and XutR/P_*xutA*_ showed the lowest basal level of expression in the absence of corresponding inducers. In contrast, MmsR/P_*mmsA*_ was found to be the leakiest of all the tested expression systems. When compared with chemical-inducible systems, such as IPTG inducible-P_*LlacO1*_ and P_*tac*_ or 3-methylbenzoate inducible-P_*m*_, our systems were shown to be equally effective (Fig. 3).

## Conclusion

Five different carbon source- or substrate-inducible promoter systems were developed for the tunable control of gene expression in *P. putida* KT2440. The use of inexpensive and amicable substances to induce promoter systems could be a feasible strategy for the large-scale production of biochemicals. Here, we report a group of promoter systems that can be induced by low-cost substrates, such as Glu, Xyl, and LA. These inducer agents can be used as potent starting materials for both cell growth and the production of a wide range of biochemicals. Expression systems such as HpdR/P_*hpdH*_ or LvaR/P_*lvaA*_ could be used to produce target biochemicals from LA as a substrate. As the xylose-inducible XutR/P_*xutA*_ system was not affected by other substrates such as Glu or LA, this system could be efficiently used to produce biochemicals from xylose. This could even occur along with Glu or LA supplemented as a carbon source(s) or co-substrate(s). To produce a wide range of chemicals from glucose, the HexR/P_*zwf1*_ system could be used. Another 3HP-inducible system (MmsR/P_*mmsA*_) did not seem to be regulated, resulting in similar GFP expression levels with or without 3HP or LA. The selection of the expression system depends on the target product, synthetic pathway, and substrate. As the efficiency of the reported systems was comparable with that of conventional chemical inducible systems, their limitations such as high cost, cell toxicity, and difficulties in downstream processes could be avoided. Hence, the newly investigated promoter systems have advantages for industrial and medical applications in *P. putida* KT2440.

## Methods

The wild-type *P. putida* KT2440 strain was used to analyze the efficiency of inducible promoter systems. Five different promoter systems —(i) HexR regulating P_*zwf1*_; (ii) LvaR regulating P_*lvaA*_; (iii) HpdR regulating P_*hpdH*_; (iv) MmsR regulating P_*mmsA*_; and (v) XutR regulating P_*xutA*_— were analyzed by expressing an enhanced GFP (eGFP^+^) under the control of each promoter system and induced by the corresponding inducer. In addition, to compare the efficiency of the constructed systems, commonly used P_*LlacO1*_, P_*tac*_, and P_*m*_ promoter systems were constructed. All promoter systems were constructed uniformly using pPROBE_P_*yqjFmut*__eGFP^+^ (pBBR1-*ori*, Km^R^: a broad-host-range expression vector). All plasmids had the same 5' untranslated region (UTR) of the pPROBE plasmid. Each constructed plasmid was transformed into the electrocompetent cells of *P*. *putida* KT2440 for the GFP assay. The details of the constructed strains and plasmids, as well as selected promoter systems and their sources for PCR amplification, are provided in Supplementary Tables S1 and S2, respectively. The oligonucleotides used for PCR amplification of each promoter system are listed in Supplementary Table S3.

The recombinant strains were cultured in Luria–Bertani medium (5 g yeast extract, 10 g peptone, and 10 g NaCl per liter) at 30 °C and 200 rpm overnight. Then, they were subcultured (initial optical density at 600 nm [OD_600_] set to 0.1) in 20 mL of M9Y medium. When the OD_600_ reached approximately 0.4, each culture (180 µL) was inoculated into a clear bottom Corning 96-well plate containing different concentrations of the corresponding inducers; they were then incubated at 30 °C with shaking in a microplate fluorescence reader (Infinite F200 PRO, Tecan, Grődig, Austria) to measure the GFP fluorescence intensity (gain of 30 at a wavelength of 485/535 nm) of the constructed systems. Fluorescence intensity was normalized based on the OD_600_ value of the culture. Subsequently, the culture was diluted appropriately with phosphate-buffered saline, and flow cytometry analysis of GFP fluorescence was performed by fluorescence-activated cell sorting (FACSCalibur Flow Cytometer, BD Bioscience, CA, USA). Approximately 2 × 10^5^ cells were analyzed per sample. The inducers and their concentrations tested for each promoter system are listed in Supplementary Table S4. All data represent the mean of two different experiments. Data were subjected to statistical analysis using SPSS (Version 11) software (SPSS Inc., Chicago, IL) to determine the level of significance. The detailed methods are provided in the supplementary information.

## Supplementary Information

Supplementary Information.

## Data Availability

The datasets generated during and/or analysed during the current study are available from the corresponding author on reasonable request.
