# Supplementary material for: Inducible and tunable gene expression systems for Pseudomonas putida KT2440
Source: Sci Rep. 2021 Sep 10;11:18079. doi: 10.1038/s41598-021-97550-7 (PMC8433446; doi:10.1038/s41598-021-97550-7)
Supplement: Supplementary file 1 — Supplementary Information. [file 41598_2021_97550_MOESM1_ESM.pdf]

Inducible and tunable gene expression systems for *Pseudomonas putida* KT2440

Chandran Sathesh-Prabu[1], Rameshwar Tiwari[1], Doyun Kim[2], and Sung Kuk Lee[1,3*]

[1]School of Energy & Chemical Engineering, Ulsan National Institute of Science and Technology (UNIST), Ulsan 44919, Republic of Korea

[2]Department of Biomedical Engineering, Ulsan National Institute of Science and Technology (UNIST), Ulsan 44919, Republic of Korea

[3]Department of Energy Engineering, Ulsan National Institute of Science and Technology (UNIST), Ulsan 44919, Republic of Korea

***Corresponding author:** sklee@unist.ac.kr

Methods

Microbial strains and plasmids

The wild-type *P. putida* KT2440 strain was used to analyze the efficiency of inducible promoter systems. The *E. coli* strain DH10B was used for cloning. The constructed strains and plasmids are listed in Supplementary Table S1.

Construction of inducible expression systems

Restriction enzymes, DNA ligase, Q5 high-fidelity DNA polymerase, and Gibson assembly cloning kit were purchased from New England Biolabs (Ipswich, MA) and used for cloning and plasmid construction. Five different promoter systems, including glucose-inducible HexR/P_{zwf1}, LA-inducible LvaR/P_{lvaA}, 3HP-inducible HpdR/P_{hpdH} and MmsR/P_{mmsA}, and xylose-inducible XutR/P_{xutA}, were constructed. To construct the inducible expression systems, fragments containing the coding sequence of the regulator of interest and the intergenic region between this gene and the translational start site of the first gene regulated by such regulators were generated by PCR using either genomic DNA of *P. putida* KT2440 or *P. fluorescens* SBW25 organisms or plasmids as templates. Next, each fragment was cloned upstream of $eGFP^{+}$ in pPROBE_$P_{yqjFmut}$_$eGFP^{+}$ at *Eco*RI/*Bam*HI sites, either by Gibson assembly or conventional cloning using restriction and ligation enzymes. In addition, to compare the efficiency of the constructed systems, commonly used P_{LlacO1}, P_{tac}, and P_{m} promoter systems were constructed. Wild-type *P. putida* KT2440 does not contain the transcription factor LacI. Therefore, to regulate P_{LacO1} or P_{tac} via the *lacO* operator sequence, LacI/LacIqP was cloned into the expression construct. To construct LacI/P_{tac}, following the amplification of LacI/LacIqP and P_{tac}/$eGFP^{+}$ from pPROBE_LacI/P_{LacO1}_$eGFP^{+}$, both fragments were cloned into pPROBE_$P_{yqjFmut}$_$eGFP^{+}$. All promoter systems were constructed uniformly using pPROBE_$P_{yqjFmut}$_$eGFP^{+}$ (pBBR1-*ori*, Km^{R}: a broad-host-range expression vector). All plasmids had the same 5' untranslated region (UTR) of the pPROBE plasmid. The details of the selected promoter systems and their sources for PCR amplification are provided in Supplementary Table S2. The oligonucleotides used for PCR amplification of each promoter system are listed in Supplementary Table S3.

Promoter expression assay

The induction of each promoter was analyzed by estimating the fluorescence intensity of the strains with different inducers. The electro-competent cells of *P. putida* KT2440 were prepared as previously described.[1] The constructed plasmid were transformed into electro-competent cells by electroporation (0.1 cm gap cuvette at a voltage of 1.8 kV) using a MicroPulser electroporator (Bio-Rad) to yield the strains HRZ01, LRL01, HRH01, MRM01, XRX01, LIL01, LIT01, and XSM01 (Supplementary Tables S1).

The inducers tested for each promoter system are listed in Supplementary Table S4. The expression assay was carried out using the following concentrations of inducers: 0, 0.5, 1, 2, 5, 10, and 20 mM Glu; and 0, 0.1, 0.2, 0.5, 1, 2, 5, 10, and 20 mM LA, 3HP, or Xyl. To analyze the cross-reactivity of inducers with the promoter systems, 10 mM of each inducer was used. IPTG (0.5 mM) for P_{LlacO1} or P_{tac} and 3MB (0.5 mM) for P_m were used. All chemicals were purchased from Sigma-Aldrich (St. Louis, MO, USA). LA was neutralized with 10N NaOH and sterile-filtered prior to use.

The recombinant strains were cultured in Luria-Bertani medium (5 g yeast extract, 10 g peptone, and 10 g NaCl per liter) at 30 °C under aerobic conditions with agitation in an orbital incubator shaker at 200 rpm overnight. Then, they were subcultured (initial optical density at 600 nm [OD_{600}] set to 0.1) in 20 mL of M9Y medium. M9Y medium contained 6.78 g NaH_2PO_4, 3 g KH_2PO_4, 0.5 g NaCl, 1 g NH_4Cl, 0.49 g $MgSO_4.7H_2O$, 0.011 g $CaCl_2$, and 5 g yeast extract per liter. Kanamycin (50 mg/L) was added to the growth medium for the cultivation of all recombinant strains. When the OD_{600} reached approximately 0.4, 180 µL of each culture was aseptically inoculated into a clear bottom Corning 96-well plate containing different concentrations of the corresponding inducers (20 µL). They were then incubated at 30 °C with shaking in a microplate fluorescence reader (Infinite F200 PRO, Tecan, Grődig, Austria) to measure the GFP fluorescence intensity (gain of 30 at a wavelength of 485/535 nm) of the constructed systems. Fluorescence intensity was normalized based on the OD_{600} value of the culture. Subsequently, the culture was diluted appropriately with phosphate-buffered saline, and flow cytometry analysis of GFP fluorescence was performed by fluorescence-activated cell sorting (FACSCalibur Flow Cytometer, BD Bioscience, CA, USA). Approximately 2×10^5 cells were analyzed per sample. All data represent the mean of two different experiments. For efficiency comparison studies, fluorescence was calculated by subtracting the basal fluorescence of the empty plasmid. Data were subjected to one-way analysis of variance

(ANOVA) or multivariate analysis of variance (MANOVA) using SPSS (Version 11) software (SPSS Inc., Chicago, IL) to determine the level of significance. *P* values less than 0.05 ($P < 0.05$) were considered significant.

Supplementary Table S1. Strains and plasmids used in this study

Strains and plasmids	Description	Ref.
**Strains**		
E. coli	DH10B; Cloning host (F⁻ *mcrA* Δ(*mrr-hsdRMS-mcrBC*) φ80*lacZ*ΔM15 Δ*lacX74 recA1 endA1 araD139* Δ(*ara-leu*)*7697 galU galK* λ⁻ *rpsL*(Str^R) *nupG*)	Lab stock
P. putida	KT2440; Wild type	Lab stock
HRZ01	KT2440 harboring pHRZ-eGFP$^+$	This study
LRL01	KT2440 harboring pLRL-eGFP$^+$	This study
HRH01	KT2440 harboring pHRH-eGFP$^+$	This study
MRM01	KT2440 harboring pMRM-eGFP$^+$	This study
XRX01	KT2440 harboring pXRX-eGFP$^+$	This study
LIL01	KT2440 harboring pLIL-eGFP$^+$	This study
LIT01	KT2440 harboring pLIT-eGFP$^+$	This study
XSM01	KT2440 harboring pXSM-eGFP$^+$	This study
**Plasmids**		
pPROBE_$P_{yqjFmut}$_eGFP$^+$	pBBR1-*ori*, Km^R	[2]
pSEVA258_*rec2*	*oriV* (RSF1010); Km^R	[3]
pBbB6c_GFP	pBBR1-*ori*, Cm^R	[4]
pHRZ-eGFP$^+$	HexR/P_{zwf1} cloned into pPROBE_$P_{yqjFmut}$_eGFP$^+$	This study
pLRL-eGFP$^+$	LvaR/P_{lvaA} cloned into pPROBE_$P_{yqjFmut}$_eGFP$^+$	This study
pHRH-eGFP$^+$	HpdR/P_{hpdH} cloned into pPROBE_P_{yqjFmu}t_eGFP$^+$	This study
pMRM-eGFP$^+$	MmsR/P_{mmsA} cloned into pPROBE_$P_{yqjFmut}$_eGFP$^+$	This study
pXRX-eGFP$^+$	XutR/P_{xutA} cloned into pPROBE_$P_{yqjFmut}$_eGFP$^+$	This study
pLIL-eGFP$^+$	LacI/P_{LlacO1} cloned into pPROBE_$P_{yqjFmut}$_eGFP$^+$	This study
pLIT-eGFP$^+$	LacI/P_{tac} cloned into pPROBE_$P_{yqjFmut}$_eGFP$^+$	This study
pXSM-eGFP$^+$	XylS/P_m cloned into pPROBE_$P_{yqjFmut}$_eGFP$^+$	This study

Supplementary Table S2. Details of the selected promoter systems constructed in this study

Promoter systems	Sources for amplification
HexR/P_{zwf1}	*P. putida* KT2440
LvaR/P_{lvaA}	*P. putida* KT2440
HpdR/P_{hpdH}	*P. putida* KT2440
MmsR/P_{mmsA}	*P. putida* KT2440
XutR/P_{xutA}	*P. fluorescens* SBW25[#]
LacI/P_{LlacO1}	pBbB6c_GFP[4]
LacI/P_{tac}	Elmore *et al.* (2017)[5]
XylS/P_m	pSEVA258_*rec2*[3]

[#]The genomic DNA of *P. fluorescens* SBW25 was a gift from Prof. Paul Rainey, Max Planck Institute for Evolutionary Biology. pSEVA258_*rec2* was a gift from Prof. Victor de Lorenzo, Centro Nacional de Biotecnología, CSIC.

Supplementary Table S3: Oligomers used in this study

Oligomers	Sequences (5’-3’)	Purpose
HexR-FP	ccggggaattctcagttgaggtcgtcgtcct	To amplify HexR/P_{zwf1} fragment from the genomic DNA of *P. putida* KT2440
P_{Zwf1}-RP	ccgggggatccccaaacagggcaaaggtgca	
LvaR-FP	ttcaggaattctcaattggcagatcgcaagc	To amplify LvaR/P_{lvaA} fragment from the genomic DNA of *P. putida* KT2440
P_{lvaA}-RP	atcggatccggttctgtaggccctgcctt	
HpdR_FP	ttcaggaattcataaaacgaaaggctcagtcg	To amplify HpdR/P_{hpdH} fragment from the genomic DNA of *P. putida* KT2440
P_{hpdH}-RP	atcggatccgtgcaacctcgcgcctgtttttattcttgtcc	
MmsR-FP	ggggatcggaagctgaattcaattcataaaacgaaaggctcagtcgaa<br>agactgggcctttcgttttatgagctctcagtcctgggcaaagcgca	To amplify MmsR/P_{mmsA} fragment from the genomic DNA of *P. putida* KT2440
P_{mmsA}-RP	ttaagcggctgcagtcgacggatcccgttctccttggaattgttgtcg	
XutR-FP	gaattggggatcggaagctgaattcctaggccgcaccctgctg	To amplify XutR/P_{xutA} fragment from the genomic DNA of *P. fluorescens* SBW25
P_{xutA}-RP	ctgcagtcgacggatccggcgttttccttattgttcttggcg	
LacI-FP	gaattggggatcggaagctgacgtcataaaacgaaaggctcagtcgaa<br>agactgggcctttcgttttatgagctctcactgcccgctttccagtc	To amplify LacI/P_{LlacO1} fragment from pBbB6c_GFP
P_{LlacO1}-RP	ttaagcggctgcagtcgacggatccatgtatatctccttcttaaaagat<br>cttttgaattcg	
LacI-P_{tac}_FP	gaattggggatcggaagctgaattctgacgtcataaaacgaaag	To amplify LacI/P_{lacI} /P_{tac} fragment from pPROBE_LacI/P_{LacO1}_eGFP^{+}
LacI-P_{tac}-RP	gatcacattatacgagccgatgattaattgtcaatagtaatcatacctgac<br>ctccatagctcgatcctctacgccggac	
P_{tac}-eGFP^{+}-FP	tcggctcgtataatgtgatcagacctggaattgtgagcggataacaattct<br>taagattaactcacacaggagatatcatggatccgtcgactgcagccg	To amplify P_{tac}/eGFP+ from pPROBE_LacI/P_{LacO1}_eGFP^{+}
P_{tac}-eGFP^{+}-RP	agtccaagctcagctaattaagcttatttgtagagctcatccatg	
XylS-FP	gaattggggatcggaagctgaattctcaagccacttcctttttg	To amplify XylS/P_{m} fragment from the pSEVA258-*rec2*
P*m*-RP	ttaagcggctgcagtcgacggatccggtatattctccttctgaattc	

Supplementary Table S4: Details of the inducers used for the different promoter systems

Promoter systems	Tested Inducers
HexR/P*zwf1*	Glu, Xyl, LA, 4HV, 3HP, mixture of GX, GLA, G4HV, G3HP
LvaR/P*lvaA*	Glu, Xyl, LA, 4HV, 3HP, mixture of GLA, XLA, LAHV
HpdR/P*hpdH*	Glu, Xyl, LA, 4HV, 3HP, mixture of G3HP, X3HP, LA3HP, 4HV3HP, GLA, XLA, LAHV
MmsR/P*mmsA*	LA, 3HP
XutR/P*xutA*	Glu, Xyl, Ara, Man, LA, 4HV, 3HP, mixture of GX, XLA, X3HP, X4HV
LacI/P*LlacO1* or P*tac*	IPTG
XylS/P*m*	3MB

Glu, glucose; Xyl, xylose; LA, levulinic acid; 4HV, 4-hydroxyvaleric acid; 3HP, 3-hydroxypropionic acid; Ara, arabinose; Man, mannose; GX, Glu+Xyl; GLA, Glu+LA; G4HV, Glu+4HV; G3HP, Glu+3HP; XLA, Xyl+LA; X3HP, Xyl+3HP; LA3HP, LA+3HP; 4HV3HP, 4HV+3HP; LAHV, LA+4HV; X4HV, Xyl+4HV; IPTG, isopropyl-β-D-thiogalactopyranoside; 3MB, 3-methylbenzoate.

References

1. Luo, X. *et al. Pseudomonas putida* KT2440 markerless gene deletion using a combination of λ Red recombineering and Cre/ *loxP* site-specific recombination. *FEMS Microbiol. Lett.* **363**, fnw014 (2016).
2. Kim, S. *et al.* Fluorescence Enhancement from nitro-compound-sensitive bacteria within spherical hydrogel scaffolds. *ACS Appl. Mater. Interfaces* **11**, 14354–14361 (2019).
3. Ricaurte, D. E. *et al.* A standardized workflow for surveying recombinases expands bacterial genome-editing capabilities. *Microb. Biotechnol.* **11**, 176–188 (2018).
4. Lee, T. S. *et al.* BglBrick vectors and datasheets: A synthetic biology platform for gene expression. *BMC Biotechnol.* **2**, 20 (2002).
5. Elmore, J. R., Furches, A., Wolff, G. N., Gorday, K. & Guss, A. M. Development of a high efficiency integration system and promoter library for rapid modification of *Pseudomonas putida* KT2440. *Metab. Eng. Commun.* **5**, 1–8 (2017).

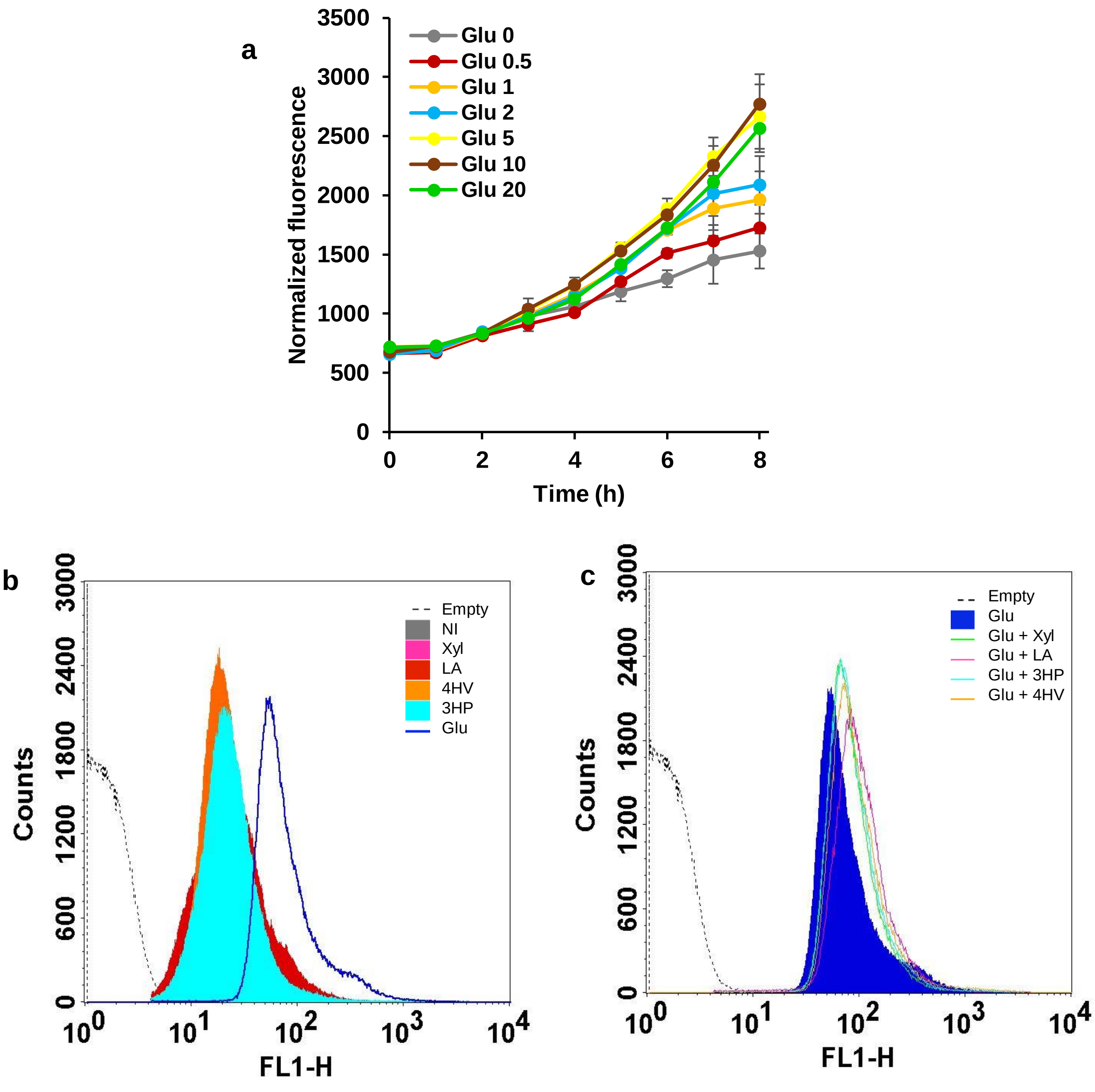

Supplementary Figure S1. Properties of the HexR/P_{zwf1} system. (a) Normalized GFP fluorescence of the system induced with different concentrations of Glu. The number after Glu refers to the concentration (mM) of Glu used. (b) Cross-reactivity of different inducers (10 mM each) with the HexR/P_{zwf1} system. Filled peaks represent the fluorescence induced by inducers other than Glu, whereas the solid line peak indicates the fluorescence induced by Glu. (c) Intactness of the HexR/P_{zwf1} system. The filled dark blue peak indicates the fluorescence of the system induced by Glu. Other solid line peaks indicate the fluorescence induced with several combinations of potential inducers (10 mM each). Glu, glucose; Xyl, xylose; LA, levulinic acid; 4HV, 4-hydroxyvalerate; 3HP, 3-hydroxypropionic acid; NI, no inducer.

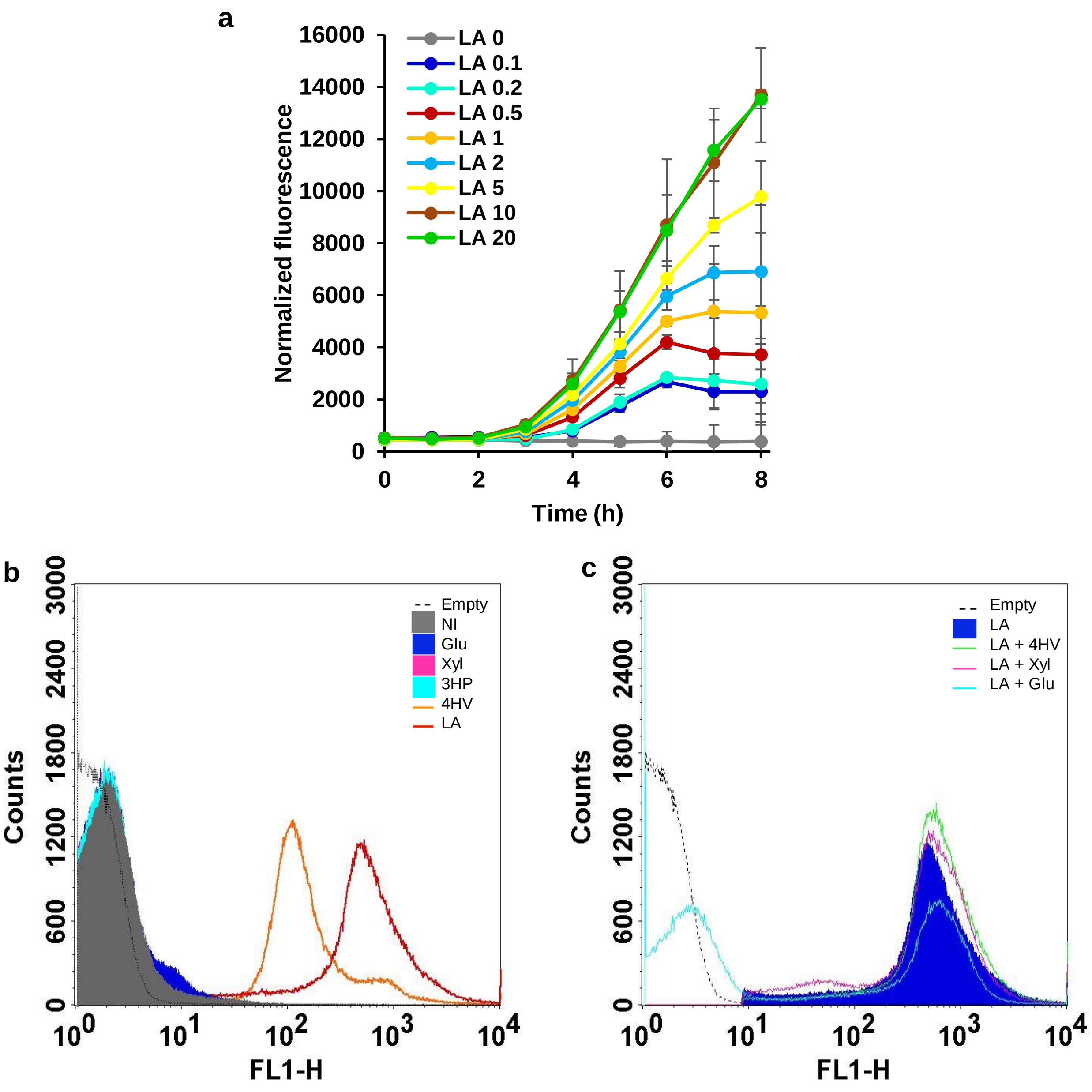

Supplementary Figure S2. Properties of the LvaR/P_{lvaA} system. (a) Normalized GFP fluorescence of the system induced with different concentrations of LA. The number after LA refers to the concentration (mM) of LA used. (b) Cross-reactivity of different inducers (10 mM each) with the LvaR/P_{lvaA} system. Filled peaks represent the fluorescence induced by inducers other than LA, whereas the solid line peaks indicate the fluorescence induced by LA and 4HV. (c) Intactness of the LvaR/P_{lvaA} system. The filled dark blue peak indicates the fluorescence of the system induced with LA. Other solid line peaks indicate the fluorescence induced with several combinations of potential inducers (10 mM each). Glu, glucose; Xyl, xylose; LA, levulinic acid; 4HV, 4-hydroxyvalerate; 3HP, 3-hydroxypropionic acid; NI, no inducer.

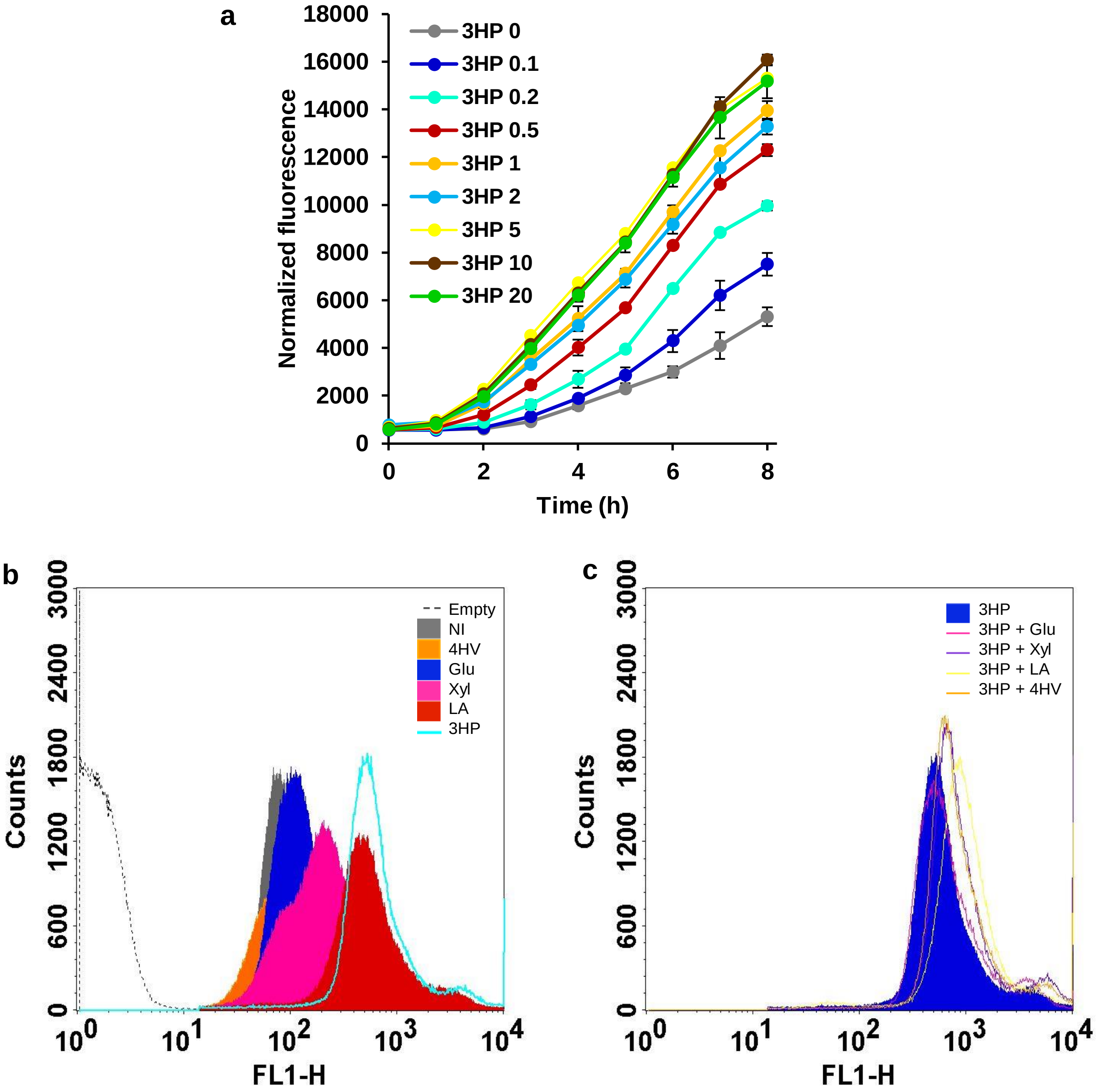

Supplementary Figure S3. Properties of the HpdR/P_{hpdH} system. (a) Normalized GFP fluorescence of the system induced with different concentrations of 3HP. The number after 3HP refers to the concentration (mM) of 3HP used. (b) Cross-reactivity of different inducers (10 mM each) with the HpdR/P_{hpdH} system. Filled peaks represent the fluorescence induced by inducers other than 3HP, whereas the solid line peak indicates the fluorescence induced by 3HP. (c) Intactness of the HpdR/P_{hpdH} system. The filled dark blue peak indicates the fluorescence of the system induced with 3HP. Other solid line peaks indicate the fluorescence induced with several combinations of potential inducers (10 mM each). Glu, glucose; Xyl, xylose; LA, levulinic acid; 4HV, 4-hydroxyvalerate; 3HP, 3-hydroxypropionic acid; NI, no inducer.

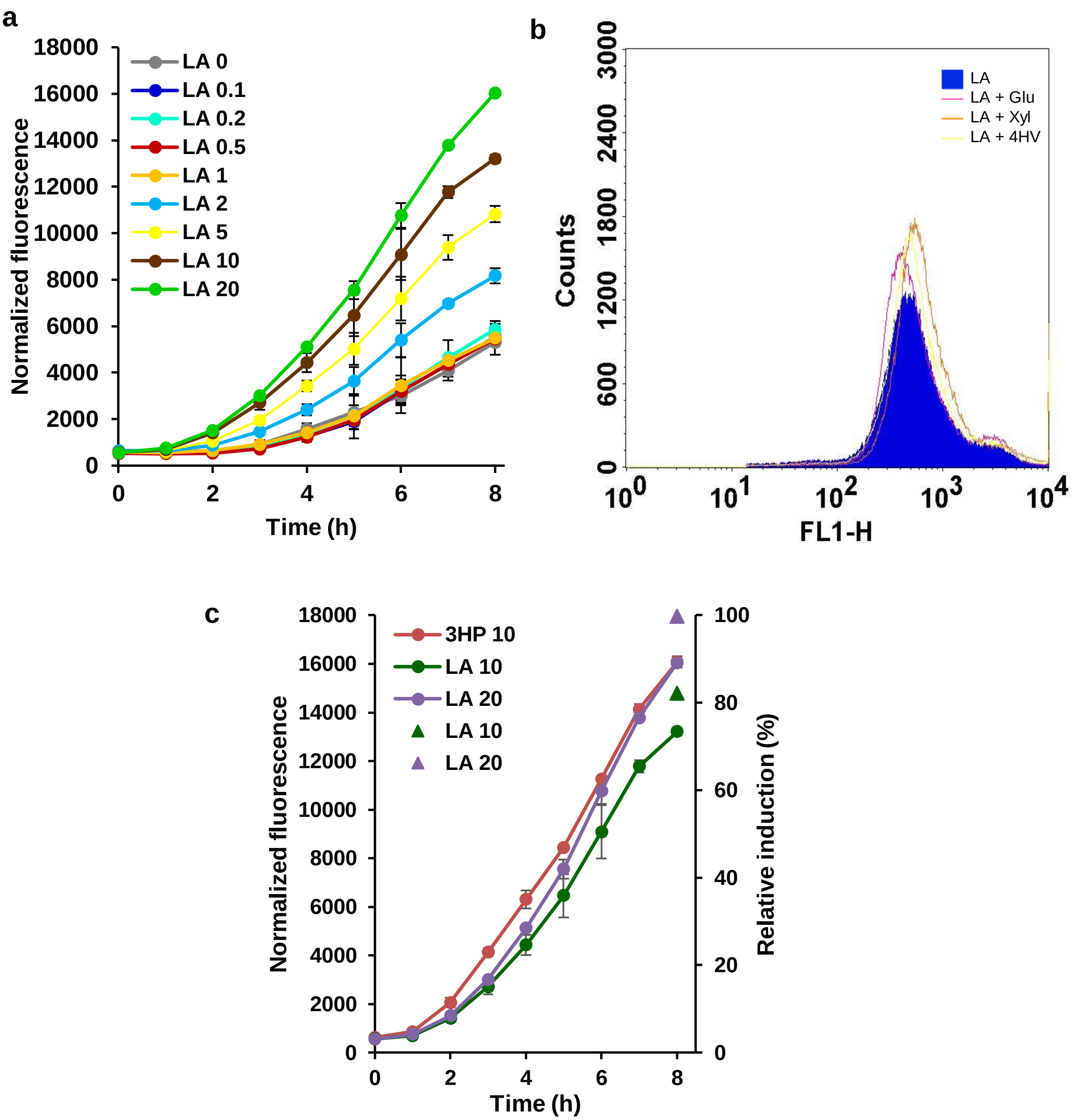

Supplementary Figure S4. Expression of the HpdR/P_{hpdH} system induced by levulinic acid. (a) Normalized GFP fluorescence of the system induced with different concentrations of LA. The number after LA refers to the concentration (mM) of LA used. (b) Intactness of the HpdR/P_{hpdH} system. The filled dark blue peak indicates the fluorescence of the system induced with LA. Other solid line peaks indicate the fluorescence induced with several combinations of potential inducers (10 mM each). (c) Relative induction level of the system by 10 mM (▲) and 20 mM (▲) of LA. Glu, glucose; Xyl, xylose; LA, levulinic acid; 4HV, 4-hydroxyvalerate.

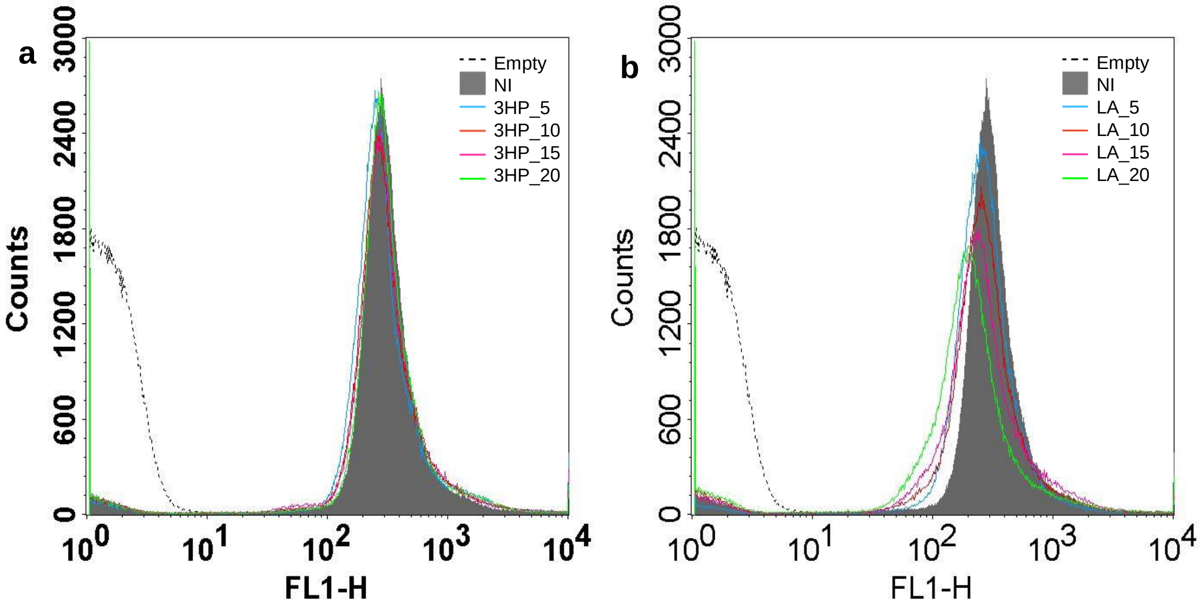

Supplementary Figure S5. Flow cytometry analysis of the MmsR/P_{mmsA} system. The system was induced with different concentrations of 3HP (a) or LA (b). The system was found to be leaky without an inducer. LA, levulinic acid; 3HP, 3-hydroxypropionic acid; NI, no inducer.

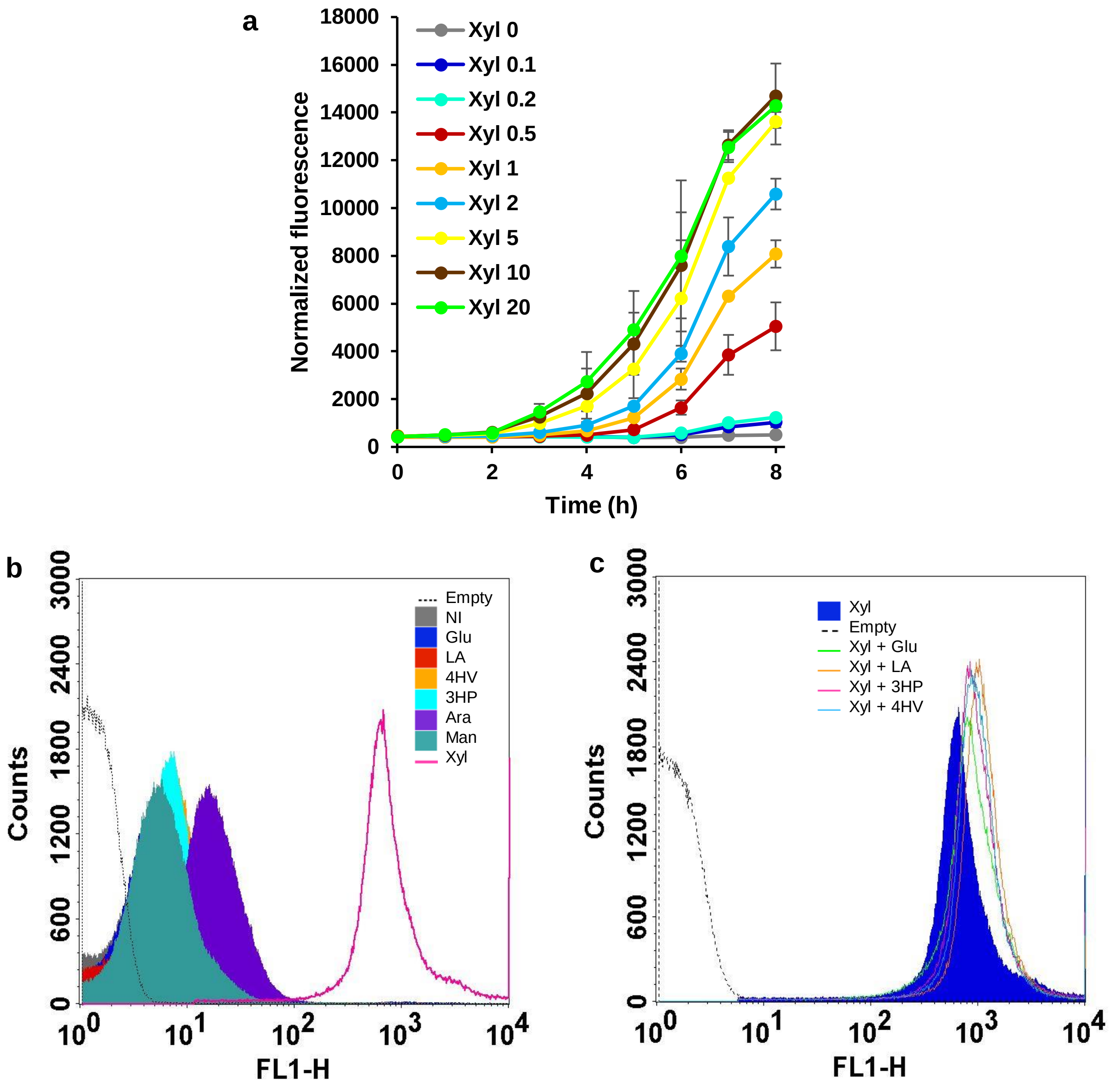

Supplementary Figure S6. Properties of the XutR/P$_{xutA}$ system. (a) Normalized GFP fluorescence of the system induced with different concentrations of Xyl. The number after Xyl refers to the concentration (mM) of Xyl used. (b) Cross-reactivity of different inducers (10 mM each) with the XutR/P$_{xutA}$ system. The filled peaks represent the fluorescence induced by inducers other than Xyl, whereas the solid line peak indicates the fluorescence induced by Xyl. (c) Intactness of the XutR/P$_{xutA}$ system. The filled dark blue peak indicates the fluorescence of the system induced with Xyl. Other solid line peaks indicate the fluorescence induced with several combinations of potential inducers (10 mM each). Glu, glucose; Xyl, xylose; LA, levulinic acid; 4HV, 4-hydroxyvalerate; 3HP, 3-hydroxypropionic acid; Ara, arabinose; Man, mannose; NI, no inducer.
